# Comparative Analysis of Secretomes from Ectomycorrhizal Fungi with an Emphasis on Small-Secreted Proteins

**DOI:** 10.3389/fmicb.2016.01734

**Published:** 2016-11-02

**Authors:** Kevin Garcia, Jean-Michel Ané

**Affiliations:** ^1^Department of Bacteriology, University of Wisconsin-Madison, Madison, WI, USA; ^2^Department of Agronomy, University of Wisconsin-Madison, Madison, WI, USA

**Keywords:** ectomycorrhizal, saprotrophs, secretomics, small-secretedproteins, symbiosis, secretomes

Ectomycorrhizal (ECM) symbioses are major components of boreal and temperate forest ecosystems (Smith and Read, 2008; Clemmensen et al., 2013). Although well studied for several decades, very little is known about the molecular players involved in the establishment and maintenance of ECM symbioses (Garcia et al., 2015). Identifying the symbiont secretome is a promising way to dissect the fungal contribution to the mutualistic molecular dialog. Pellegrin et al. (2015) compared for the first time the predicted secretome of 49 soil-borne ECM, saprotrophic and pathogenic fungi, revealing shared and specific features between species, and providing a better understanding of the ECM lifestyle evolution.

## Fungal secretomes support the saprotrophic—ECM fungi continuum

Comparative genomic studies revealed that ECM fungi evolved multiple times from saprotrophic ancestors, and have partially lost their wood decay capabilities. This can be explained by the convergent loss of multiple lignin oxidoreductases, class II peroxidases and plant cell wall degradation enzymes (Martin et al., 2008, 2010; Tedersoo et al., 2010; Floudas et al., 2012; Wolfe et al., 2012; Kohler et al., 2015). The comparison of fungal secretome released by Pellegrin et al. (2015) supports this view.

Although features are shared between all studied fungi including the secreted lipases, proteases, and Small-Secreted Proteins (SSPs), ECM-specific features that support the transition toward a mutualistic lifestyle were highlighted. A reduction of Carbohydrate-Active enZymes (CAZymes) compared to other species was observed in ECM fungi, confirming a reduction of plant cell wall degradation capabilities.

Various SSPs called effectors are well known in pathogenic fungi to manipulate plant defenses to facilitate infection (Stergiopoulos and de Wit, 2009). Some SSPs with a similar activity were also described by functional analyses in mutualistic plant–fungal association (Kloppholz et al., 2011; Plett et al., 2011; Tsuzuki et al., 2016). More recently, a comparative *in silico* analysis revealed that many SSPs were shared by both arbuscular mycorrhizal fungi *Rhizophagus clarus* and *Rhizophagus irregularis*, supporting the role of secreted peptides in mycorrhizal associations (Sędzielewska Toro and Brachmann, 2016). Remarkably, Pellegrin et al. (2015) predicted more SSPs in the secretome of ECM than saprotrophic fungi, including many that are ECM-specific (17 clusters). This observation suggests that those specific SSPs could be a signature of the ECM symbiosis lifestyle and could play a predominant role in the molecular dialog with the host plants.

## Can ECM-specific SSPs be involved in early steps of mycorrhizal symbiosis formation?

Mining the genome of the first ECM fungus, *Laccaria bicolor*, allowed the prediction of SSPs potentially involved in free-living conditions, mycorrhiza formation, or both (Martin et al., 2008). Later, SSPs have been identified in the secretome of the ECM fungi *L. bicolor* and *Hebeloma cylindrosporum* cultured without their host (Vincent et al., 2012; Doré et al., 2015). Pellegrin et al. (2015) predicted SSPs that are shared by ECM, saprotrophic, and pathogenic fungi, suggesting conserved mechanisms in hyphal development, fruiting body formation, or interaction with other soil organisms and the environment. Similarly, the prediction of ECM-specific SSPs suggests that they would be part of a molecular dialog with host plants, leading to the formation of functional ECM. So far, only fungal flavonoids and plant phytohormones were described in the early stages of ECM symbiosis establishment, but lipochitooligosaccharides, chitin oligomers and even SSPs could be hypothesized as part of this initial cross-talk too (Garcia et al., 2015).

Another function that could be attributed to fungal SSPs would be their involvement in host-specificity. Although a high degree of specificity is rare in ECM forests, some fungi can be found exclusively associated with few tree species (Churchland and Grayston, 2014). Thus, secreted molecules including SSPs could facilitate the establishment of such specific interactions. It is also possible to speculate that signaling molecules found in plant root exudates might trigger the expression of fungal SSPs, allowing the initiation of a specific interaction. The identification of thousands of SSPs from various ECM fungi combined with the development of transgenic transformation technologies will provide new insights on the molecular cues and signals participating in the establishment and function of ECM associations.

## Toward the validation of ECM-specific small-secreted proteins

To this date, only one SSP named Mycorrhizal-induced Small-Secreted Protein 7 (MiSSP7) from *L. bicolor* was described in a symbiotic context. Pellegrin et al. (2015) and previous large-scale studies highlighted many other SSPs with unknown function (Martin et al., 2008; Doré et al., 2015; Kohler et al., 2015).

The generation of transgenic fungi affected in the expression of MiSSP7 was a turning point in the functional validation of ECM-specific SSPs. The down-regulation of MiSSP7 resulted in the inhibition of the Hartig net formation in poplar by interfering with jasmonic acid immune response (Plett et al., 2011, 2014). Excitingly, some other ECM fungi studied by Pellegrin et al. (2015) are also transformable including *H. cylindrosporum* (Combier et al., 2003) and *Pisolithus tinctorius* (Rodríguez-Tovar et al., 2005). This technology opens the way to further systematic validations using RNA interference, CRISPR/Cas9, or over-expression approaches in both free-living and symbiotic conditions (Garcia et al., 2014; Xu et al., 2015). It is worth noting that *P. tinctorius* might become a particularly interesting model to understand the role of ECM-specific SSPs. Among the 17 ECM-specific SSP clusters, *P. tinctorius* SSPs were found in 12 of them.

Other approaches based on the host plant can also be envisioned to understand the role of ECM-specific SSPs including the application of purified SSPs on plant roots, or the ectopic expression of SSPs in genetically transformable hosts like poplar. Finally, identifying plant genes targeted by fungal SSPs using for example yeast-two-hybrid experiments will be ultimately needed to unravel the function of those small molecules in symbiotic associations.

## Author contributions

The manuscript was written and edited by KG and J-MA.

## Funding

KG was supported by a grant from the National Science Foundation (NSF-IOS#1331098) to J-MA.

### Conflict of interest statement

The authors declare that the research was conducted in the absence of any commercial or financial relationships that could be construed as a potential conflict of interest.
